# Assessing general cognitive and adaptive abilities in adults with Down syndrome: a systematic review

**DOI:** 10.1186/s11689-019-9279-8

**Published:** 2019-08-30

**Authors:** Sarah Hamburg, Bryony Lowe, Carla Marie Startin, Concepcion Padilla, Antonia Coppus, Wayne Silverman, Juan Fortea, Shahid Zaman, Elizabeth Head, Benjamin L. Handen, Ira Lott, Weihong Song, André Strydom

**Affiliations:** 1 Department of Forensic and Neurodevelopmental Sciences, Institute of Psychiatry, Psychology & Neuroscience, Kings College London, London, SE5 8AF UK; 2 Division of Psychiatry, University College London, London, W1T 7NF UK; 3 The London Down Syndrome Consortium (LonDownS), London, UK; 4 Department of Psychology, Loughborough University, Loughborough, Leicestershire LE11 3TU UK; 5 Department of Psychiatry, Herchel Smith Building for Brain & Mind Sciences, Forvie Site, Robinson Way, Cambridge, CB2 0SZ UK; 6 Dichterbij, Center for Intellectual Disabilities, Gennep, The Netherlands; 7 Department of Primary and Community Care, Radboud University Medical Center, Nijmegen, The Netherlands; 8 Department of Pediatrics, University of California, Irvine, USA; 9 Memory Unit, Department of Neurology, Hospital de la Santa Creu i Sant Pau–Biomedical Research Institute Sant Pau, Universitat Autònoma de Barcelona, Barcelona, Spain; 10 Barcelona Down Medical Center, Fundació Catalana de Síndrome de Down, Barcelona, Spain; 11 Centro de Investigación Biomédica en Red de Enfermedades Neurodegenerativas, CIBERNED, Madrid, Spain; 12 Sanders-Brown Center on Aging, University of Kentucky, 800 South Limestone Street, Lexington, KY 40536-0230 USA; 13 Department of Psychiatry, University of Pittsburgh School of Medicine, Pittsburgh, PA 15213 USA; 14 Departments of Pediatrics and Neurology, University of California, Irvine, USA; 15 Townsend Family Laboratories, Department of Psychiatry, The University of British Columbia, 2255 Wesbrook Mall, Vancouver, BC V6T 1Z3 Canada

**Keywords:** Down syndrome, Cognition, Intelligence, IQ, Adaptive behaviour, Adaptive ability, AB, General ability

## Abstract

**Background:**

Measures of general cognitive and adaptive ability in adults with Down syndrome (DS) used by previous studies vary substantially. This review summarises the different ability measures used previously, focusing on tests of intelligence quotient (IQ) and adaptive behaviour (AB), and where possible examines floor effects and differences between DS subpopulations. We aimed to use information regarding existing measures to provide recommendations for individual researchers and the DS research community.

**Results:**

Nineteen studies reporting IQ test data met inclusion for this review, with 17 different IQ tests used. Twelve of these IQ tests were used in only one study while five were used in two different studies. Eleven studies reporting AB test data met inclusion for this review, with seven different AB tests used. The only AB scales to be used by more than one study were the Vineland Adaptive Behaviour Scale (VABS; used by three studies) and the Vineland Adaptive Behavior Scale 2nd Edition (VABS-II; used by two studies). A variety of additional factors were identified which make comparison of test scores between studies problematic, including different score types provided between studies (e.g. raw scores compared to age-equivalent scores) and different participant inclusion criteria (e.g. whether individuals with cognitive decline were excluded). Floor effects were common for IQ tests (particularly for standardised test scores). Data exists to suggest that floor effects may be minimised by the use of raw test scores rather than standardised test scores. Raw scores may, therefore, be particularly useful in longitudinal studies to track change in cognitive ability over time.

**Conclusions:**

Studies assessing general ability in adults with DS are likely to benefit from the use of both IQ and AB scales. The DS research community may benefit from the development of reporting standards for IQ and AB data, and from the sharing of raw study data enabling further in-depth investigation of issues highlighted by this review.

## Background

Down syndrome (DS) is the most common genetic cause of intellectual disability (ID), with an incidence of around 1 in 650–1000 live births worldwide [1]. DS occurs due to an extra copy of chromosome 21 (trisomy 21), typically in its entirety and in all cells. However, in rarer cases of DS, only some cells have an extra copy of chromosome 21 (mosaicism) or only part of chromosome 21 is triplicated by translocation (partial trisomy). People with DS may have significant cognitive impairments and typically have an intelligence quotient (IQ) ranging from 30 to 70, although IQs both above and below this range occur [2]. Cognitive domains that are particularly impaired in individuals with DS include language (especially expressive language), memory, executive function, and motor coordination. These impairments can vary substantially among individuals with DS and also within individuals due to advanced adult age and/or the development of dementia (for which people with DS are at an ultra-high risk [lifetime prevalence of dementia is estimated to be as high as 90% [3]], although considerable variability is present in terms of age at dementia onset and clinical presentation, as reviewed by Zigman and Lott [4]).

In addition to impairments in general cognitive ability, individuals with DS also have considerable limitations in adaptive behaviour (AB). Adaptive skills are defined as “the effectiveness with which the individual copes with the natural and social demands of his environment” [5]. Although they reflect distinct domains of functioning, adaptive skills/abilities are associated with general cognitive ability measured with IQ [2], suggesting AB scales may be used as an alternative for estimating the severity of ID in individuals when IQ assessment results are unavailable.

Due to the unique cognitive profile found in people with DS (see [6]), it is necessary to understand how useful and applicable different IQ tests and AB scales are for this population as an index of general abilities. Understanding the relationship between IQ and AB scores across the lifespan is also of importance as there is decline in both IQ and AB scores as people with DS age [7, 8]. This is thought to be associated with the development of Alzheimer’s disease (AD). However, other conditions such as untreated hypothyroidism or emergent neuropsychiatric symptoms, such as the development of depression, may also impact capabilities and performance during assessments. Cohort effects, such as improvements in healthcare and education (including the phasing out of institutions), are also important considerations for cross-sectional studies [9].

IQ tests and AB scales are commonly used in DS studies to describe and compare participant samples, establish the impact of interventions/treatments or comorbidities, and track cognitive change with development and ageing. Such assessments may be particularly important in clinical trials of treatments to improve cognitive outcomes or to track the trajectory of decline due to advanced age or dementia. However, assessment of general ability in individuals with DS is complicated by floor-effects for many neuropsychological tests that were developed for use within the typically developing (TD) population [10–12]. In addition, a relative weakness in language domains is often present for people with DS, which may complicate interpretation of performance on verbal tests and those with a large verbal component [13].

The aim of this systematic literature review is to summarise currently available literature on the different IQ and AB tests used previously with adults with DS, with a particular focus on direct comparisons between tests as well as differences in performance between participant groups (younger adults and older adults with and without dementia) in order to make recommendations for future studies assessing general cognitive abilities in adults with DS, and also for the wider DS research community (see Table 3).

## Systematic review methods

### Search strategy and selection criteria: IQ tests

The PubMed database was searched using the search terms (Down syndrome [MeSH Major Topic]) AND (“Intelligence Quotient” OR “IQ” [All Fields] OR “Stanford-Binet test” [MeSH Major Topic] OR “K-BIT” [All Fields] OR “BPVS” [All Fields] OR “Leiter” [All Fields] OR “Raven’s Matrices” [All Fields] OR “Wechsler scales” [MeSH Terms] OR (“Wechsler” [All Fields] AND “scales” [All Fields]) OR “Wechsler scales” [All Fields] OR “WISC” [All Fields] OR “Peabody” [All Fields] OR “WPPSI” [All Fields] OR “Otis-Lennon” [All Fields] OR “Differential Ability Scales” [All Fields] OR “Woodcock-Johnson” [All Fields]) on 23 September 2018, identifying a total of 197 papers. Titles and abstracts were first screened to identify studies meeting the following criteria for inclusion: papers were written in English (translations accepted), the study was published from 1990 onwards, and there was a minimum of 20 participants with DS aged 16 years or older included in the study. This brought the total number of eligible studies down to 75.

Full articles were then read in detail to identify which met the following additional inclusion criteria. We included tests of vocabulary, as these are often viewed as tests of general ability due to their strong correlation with IQ. Statistical data (including at least one of the following: mean, median, standard deviation, range, floor effects) from a named IQ or general ability test was provided. Where not all individuals in the study were 16 years or older or not all participants had a diagnosis of DS, papers were only included where separate statistical data (at least one of the following: mean, median, standard deviation, range, floor effects) was provided for participants with DS aged 16 years or older. This brought the total number of eligible studies down to 14. If the same or overlapping participants were used in multiple studies, we selected the main report for inclusion, discarding a further four papers. Additionally, reference lists of identified articles were examined to identify other relevant studies, adding five papers, and a further four papers were included due to knowledge of the research area. This resulted in a total of 19 relevant papers.

All available data regarding sample size, age of participants, IQ test used, performance on tests, floor effects (if available), and whether the study reported raw and/or standardised test scores was extracted from included papers.

### Search strategy and selection criteria: AB scales

For AB scales in DS, the same database (PubMed) was searched using the search terms (“Down syndrome” [MeSH Major Topic]) AND (“Adaptive Behavior Scales” [All fields] OR “Vineland” [All fields] OR “Adaptive Behavior Assessment System” [All fields] OR “Diagnostic Adaptive Behavior Scale” [All fields] OR “Adaptive Behavior” [All fields] OR “Every day abilities” [All fields] OR “Scales of Independent Behavior” [All fields] OR “Barthel Index” [All fields] OR “Wessex Behaviour Scale” [All fields]) on 23 September 2018, identifying a total of 69 papers. AB papers were included in the review using the same criteria as for the IQ papers detailed above and dropped to 36 after screening the title and abstract. After reading the full article, 28 papers were discarded. Reference lists of identified articles were examined to identify other relevant studies, adding two papers. One additional paper was included due to knowledge of the research area. This left a total of 11 relevant papers.

All available data regarding sample size, age of participants, AB scale used, test performance, floor effects (if available), and whether the study reported raw and/or standardised test scores was extracted from included papers.

## IQ and general ability tests in people with DS

### Tests

Nineteen studies, comprising 1455 participants (range 26–305 participants), meeting inclusion criteria that reported IQ or general ability test scores are shown in Table 1. A wide range of ages are included in this review, with the oldest participant being 71 years old. A brief description of all tests identified within this review is provided in the Appendix Table 4.

**Table 1:** Summary of studies using intelligence tests in adults with DS. Tests are arranged into those not specifically designed for children and adolescents and those that are. AB tests not shown (see Table 2). Ages and age-equivalents given in years; where given in months in original papers these have been converted. Ages and scores given as mean (SD; range). NR indicates “not reported”

Study	IQ test	Score type(s) provided	Participants	Participant ages	Raw scores	Standardised scores	Floor effects
Tests not specifically designed for children and adolescents (most recent first)							
Lao et al., [26]	PPVT-IV	Standardised score and age-equivalent score	52	37.3 (6.6; 30–50)	NR	56.6 (17.2) standardised score; 8.19 (3.44) age-equivalent score	NR
Hartley et al. [14]*	PPVT-IV	Age-equivalent score	58	37.6 (6.8; ≥ 30)	NR	8.10 (3.34)	NR
Tomaszewski et al. [15]	Stanford Binet 5th Ed	Full IQ score	31	25.9 (5.92)	NR	46.6 (9.1)	NR
Sinai et al. [11]	KBIT-2	Raw scores for verbal and non-verbal subscales	30 no dementia	50.9 (4.83)	Total, 23.17 (19.50; 3–63); Verbal, 16.37 (13.33; 1–47); Non-verbal, 6.8 (6.92; 0–15)	NR	Verbal, 0%; Non-verbal, 16.7%
			19 diagnosed or possible dementia	55.6 (6.77)	Total, 9.74 (11.06; 1–49); Verbal, 6.53 (7.16; 0–34); Non-verbal, 3.21 (4.16; 0–20)	NR	Verbal, 5.3%; Non-verbal, 21.1%
Startin et al. [16]	KBIT-2	Raw scores for verbal and non-verbal subscales; full IQ scores floor effects only	130 aged 36+ years without dementia	47.77 (7.01; 36–71)	Verbal, 30.55 (17.47; 2–80); Non-verbal, 12.55 (6.57; 0–32);	NR	Verbal raw, 0%; Verbal IQ, 66.7%; Non-verbal raw, 6.7%; Non-verbal IQ, 39.4%
			51 aged 36+ years with dementia	54.20 (6.95; 38–67)	Verbal, 18.68 (13.77; 1–51); Non-verbal, 8.29 (6.45; 0–19)	NR	Verbal raw, 0%; Verbal IQ, 84.0%; Non-verbal raw, 16.7%; Non-verbal IQ, 62.5%
			124 aged 16–35 years	25.24 (5.53; 16–35)	Verbal, 35.03 (16.77; 2–82); Non-verbal, 14.98 (6.9; 0–32)	NR	Verbal raw, 0%; Verbal IQ, 50.8%; Non-verbal raw, 4.1%; Non-verbal IQ, 33.9%
de Sola et al. [17]	KBIT (Spanish version)	Full IQ score; combined verbal and non-verbal standardised KBIT score	86	23.3 (4.3; 16–34)	NR	Full IQ median, 41; Standardised KBIT score, 105 (17.8; 80–180)	41.9%
Ghezzo et al. [8]	WAIS-R	Full IQ score; verbal IQ score; performance IQ score	36 adults with DS (of a larger sample of 67 participants which included children)	18–29 years.: *n* = 24, 22.34 (3.40) 30–39 years.: *n* = 17, 34.27 (3.04) ≥ 40: years. *n* = 18, 49.34 (6.91)	NR	Total IQ 18–29 years., 49.71 (12.69) 30–39 years., 48.80 (11.84) ≥ 40, 33.20 (19.60) Verbal IQ 18–29 years., 53.43 (13.02) 30–39 years., 51.60 (12.91) ≥40, 33.60 (20.02) Performance IQ 18–29 years., 51.38 (12.49) 30–39 years., 52.90 (12.44) ≥40, 36.20 (23.72)	NR
Breia et al. [18]	WAIS-III (Portuguese version)	Full IQ score; verbal IQ score; non-verbal IQ score	26 (of a larger sample of 209)	Full sample, 32.6 (8.58)	NR	Full scale IQ, 49.65 (4.93; 45–61); Verbal IQ, 52.27 (5.65; 45–64); Non-verbal IQ, 50.77 (5.06; 45–62)	NR
Iacono et al. [13]	PPVT-III	Age-equivalent score	55	38 (19–58)	NR	5.17 (2.17; 1.67–9.75)	NR
	RCPM	Raw score	55	38 (19–58)	10.65 (3.95; 4–20)	NR	NR
Kay et al. [19]	PCFT	Raw scores	85	38.2	88.0 (61.9; 0–224) Median 97	NR	NR
Patel et al. [20]	Five subtests from the early-development battery of the WJTCA-R	Raw scores	82 females (58 pre-menopausal, 24 post-menopausal), 80 males	Total range 21–57; premenopausal females 34.7 (6.8), postmenopausal females 49.7 (4.2)	Pre-menopausal females, 468.7 (15.9); age-matched males, 462.2 (17.7) Post-menopausal females; 446.2 (19.0 SD); age-matched males, 453.1 (23.3 SD)	NR	NR
Tests designed for children and adolescents (most recent first)							
de Knegt et al. [21]	WPPSI-R	Age-equivalent score	244	38.1 (11.1)	NR	5.0 (1.5)	NR
d’Ardhuy et al. [10]	Leiter-R (full)	Non-verbal IQ score	41	22.7 (3.4; 18–30)		39.0 (6.0; 36–65)	61%
Dressler et al. [22]	RCPM** or Leiter-R	Age-equivalent score	49	28.8 (8.4; 19–52)	NR	4.72 (2.46; 3.06–10.0)	NR
Strydom et al. [23]	BPVS-II	Raw and age-equivalent score	32 (10 mild ID, 18 moderate ID, 4 severe ID)	32.59 (6.78; 18–45)	67.8 (22.89; 14–112)	Mild ID, 7.8; Moderate ID, 4.7; Severe ID, 2.04; Overall range 2.04–12.01	3 individuals could not complete the test
Glenn and Cunningham [12]	BPVS-II	Age-equivalent score	46	19.83 (1.92; 16.17–24.33)	NR	6.53 (1.98)	NR
	Leiter-R (brief)	Non-verbal IQ; age-equivalent score	46	19.83 (1.92; 16.17–24.33)	NR	Non-verbal IQ, 3.3 (0.5); Age-equivalent, 5.2 (1.0)	Majority of IQ scores were 36, with very few over 45, despite age-equivalent scores differing
Kittler et al. [24]*	WISC-R	Raw scores	42 (21 females, 21 males)	Female, 37.9 (5.9) Male, 40.3 (5.7)	Verbal subtests: Information: F 6.6 (3.7), M 7.2 (4.0) Similarities: F 4.0 (5.3), M 3.2 (4.5) Arithmetic: F 3.1 (2.0), M 2.7 (1.8) Vocabulary: F 13.9 (7.2), M 17.3 (9.1) Comprehension: F 6.7 (4.7), M 7.4 (5.0) Non-verbal subtests: Picture completion: F 7.8 (5.3), M 8.6 (4.4) Picture arrangement: F 4.4 (5.1), M 2.6 (3.6) Block design: F 9.6 (7.3), M 8.0 (6.0) Object assembly: F 11.7 (6.1), M 8.7 (5.8) Coding: F 22.0 (10.5), M 15.7 (9.6)	NR	40% scored 0 or 1 on Picture Arrangement; 48% scored 0 or 1 on Similarities
Devenny et al. [25]*	WISC-R	Subtest raw scores	44	46.85 (6.01)	Information, 6.64 (3.71); Arithmetic, 3.00 (2.03); Vocabulary, 15.59 (7.83); Comprehension, 7.17 (5.14); Picture completion, 7.67 (4.69); Block design, 8.82 (6.90); Object assembly, 9.68 (6.17); Coding, 18.33 (10.82); Digit span, 2.98 (2.25)	NR	52% scored 0 or 1 on Picture Arrangement; 66% scored 0 or 1 on Similarities
Das et al. [7]	PPVT-R**	Raw score	16 younger	43.7 (2.9; 40–49)	57.75 (21.16)	NR	NR
			16 older	55.2 (3.9; 50–62)	43.00 (40.98)	NR	NR
	MAT	Raw score	16 younger	43.7 (2.9; 40–49)	6.25 (4.67)	NR	“Too difficult for most participants”
			16 older	55.2 (3.9; 50–62)	3.75 (3.51)	NR	

*Only T1 data used in this review

**Not designed for children and adolescents

In total, 17 different IQ or general ability tests were used across the 19 identified studies. Twelve of these IQ tests were used in only one study while five were used in two different studies. These five tests were the Kaufman Brief Intelligence Test 2nd edition (KBIT-2) [11, 16], the Wechsler Intelligence Scale for Children-Revised (WISC-R) [24, 25], Raven’s Coloured Progressive Matrices (RCPM) [13, 22], the British Picture Vocabulary Scale 2nd edition (BPVS-II) [12, 23], and the Peabody Picture Vocabulary Test 4th edition (PPVT-IV) [14, 26].

In addition to this, different versions of the same test were used by a number of studies. These included the Peabody Picture Vocabulary Test-Revised (PPVT-R) and the Peabody Picture Vocabulary Test 3rd edition (PPVT-III) [7, 13], in addition to the Leiter International Performance Scale-Revised (Leiter-R) [10] and a brief version of this test [12]. The Wechsler Adult Intelligence Scale-III (WAIS-III; Portuguese version) and Wechsler Adult Intelligence Scale Revised were also each used once. Furthermore, de Sola et al. [17] used the Spanish version of the KBIT.

Five tests were used in only one study and also had no alternative versions used. These included the Prudhoe Cognitive Function Test (PCFT), the Woodcock-Johnson Tests of Cognitive Ability-Revised (WJTCA-R), the Matrix Analogies Test-Expanded Form (MAT), the Wechsler Preschool and Primary Scale of Intelligence—revised version (WPPSI-R), and the Stanford Binet 5th edition.

### Participant samples

Although some studies have used the same or different versions of the same test, comparison between studies is complicated by differing participant inclusion criteria. For example, some studies grouped participants by dementia status and provide separate test results for each group [11, 16], or only include individuals without a diagnosis of dementia or noticeable decline [8, 19, 20, 23–26], while in other studies these participants are included in the overall sample [7, 13]. Different criteria to define and/or detect dementia were also used between studies.

Furthermore, some studies restricted inclusion to more able participants. For example, “participants were required to have sufficient verbal ability to be interviewed” [20], participants were required to have “verbal oral language skills” [18], inclusion of participants with mild-moderate ID only [24], inclusion criteria of IQ > 30 [25], inclusion criteria of a mental age above 2.5 years in addition to at least minimal verbal communication [14], inclusion criteria of receptive language > 3 years. [26], and the inclusion of individuals not at floor only [13]. All such studies were still included in this review, despite differing individual inclusion criteria. Such differing criteria will substantially skew floor effects between studies and make comparison between studies problematic.

### Floor effects

Nine of the 17 studies reported data on floor effects for the IQ or general ability tests they used [10–12, 14, 16, 17, 23–25]. Additionally, floor effects were alluded to by Das et al. [7], who indicated the MAT was “too difficult for most participants”. Of the remaining studies, five studies did not report data on floor effects [15, 19, 21, 22, 26], two studies only included individuals who were able to provide a verbal response [18, 20], and one study only included individuals above floor levels [13].

Studies using standardised test scores reported particularly large floor effects. These were as high as 61% for the Leiter-R [10]. Glenn and Cunningham [12] also reported large floor effects for the brief Leiter-R (the “majority” of test scores were at floor). For the KBIT (Spanish version), de Sola et al. [17] reported floor effects of 41.9% for standardised IQ scores. When examining KBIT-2 IQ subscales independently, Startin et al. [16] reported floor effects of 66.7% for verbal IQ and 39.4% for non-verbal IQ (adults aged 36+ without a clinical diagnosis of dementia).

**Fig. 1:**
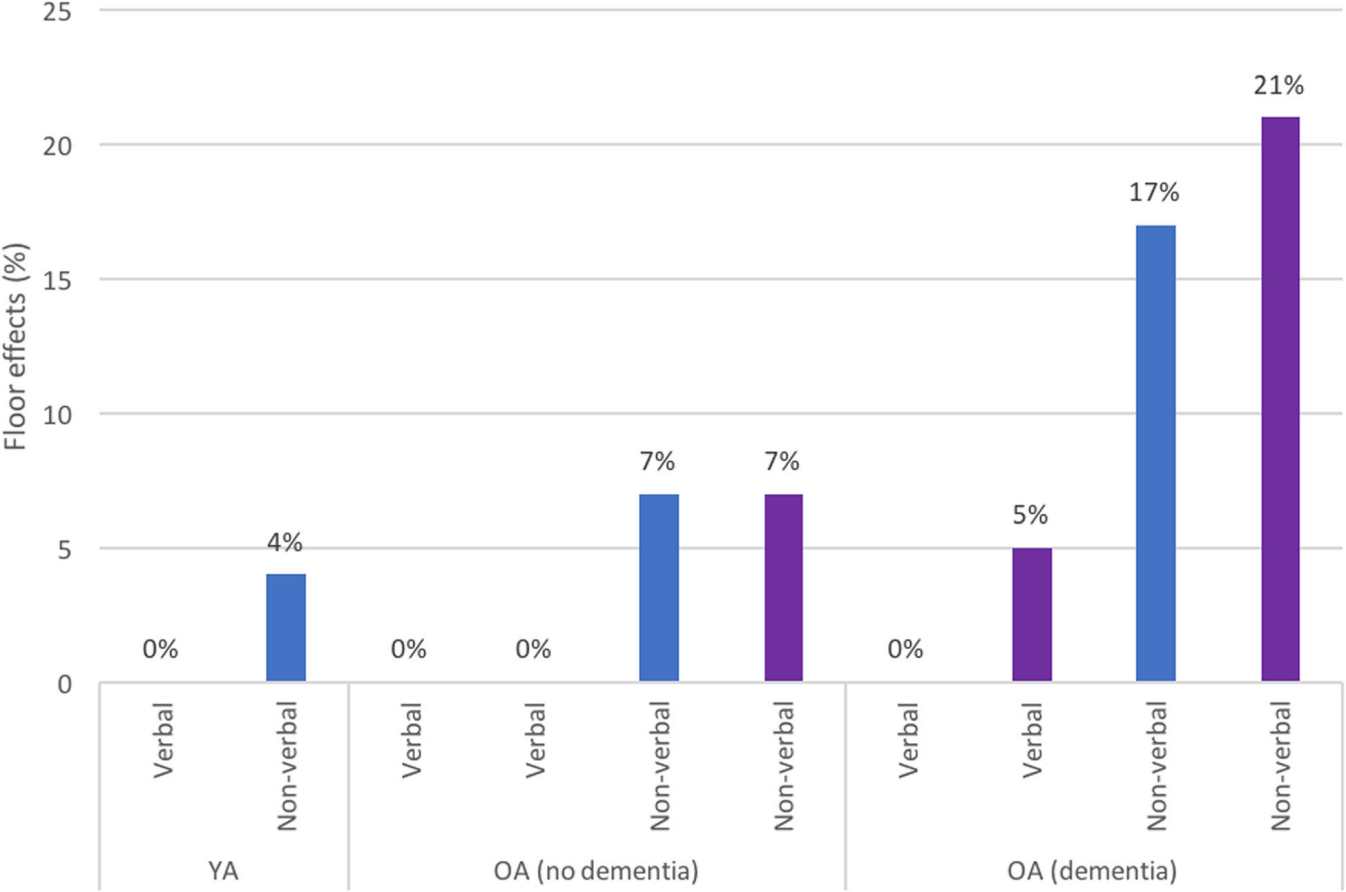
KBIT-2 floor effects. Percentage of participants at floor for KBIT-2 subscales by participant group (younger adults (YA), older adults without dementia (OA-ND), and older adults with dementia (OA-D)) for individual studies reporting these values (Startin et al. (blue) [16]; Sinai et al. (purple) [11])

For studies reporting IQ test raw scores, using the WISC-R, Kittler et al. [24] reported 40% and 48% of participants scored 0 or 1 on the first administration of the picture arrangement subtest and the similarities subtest, respectively. Devenny et al. [25] also reported high floor effects for these same subscales (52% and 66%, respectively). In contrast to this, when analysing KBIT-2 raw scores, two studies [11, 16] found no or limited floor effects for the verbal subscale (based on receptive language rather than expressive language). The KBIT-2 non-verbal subscale had moderate floor effects across both younger (YA) and older adults (OA), and these increased substantially in participants with dementia (see Fig. 1). Raw scores were also used by Strydom et al. [23] on the BPVS-II, with moderate floor effects (9.4%) reported.

### Comparison between IQ test scores

#### Age-equivalent scores

Two IQ tests were identified for which age-equivalent scores were reported by more than one study. Using the BPVS-II (which provides an estimate of receptive language), Glenn and Cunningham [12] reported a mean age-equivalent score of 6.5 years for their sample of younger adults with DS (age range 16–24 years). Strydom et al. [23] reported BPVS-II mean age-equivalent scores separately for participants with mild, moderate, and severe ID (7.8 years, 4.7 years, and 2.0 years, respectively). Interestingly, Glenn and Cunningham [12] also provided non-verbal age-equivalent scores for their participants, using the Brief Leiter-R (mean 5.2 years). Although the higher mean verbal age-equivalent score in this study compared to mean non-verbal (6.5 vs 5.2 years) is not consistent with the cognitive profile associated with DS, the difference is small and SD scores overlap.

Using the PPVT-IV (which also provides a measure of receptive language), Hartley et al. [14] reported a mean age-equivalent score of 8.1 years for their sample of adults with DS aged 30 years or older. Using the same test, Lao et al. [26] reported a mean age-equivalent score of 8.2 years for their sample of adults with DS aged 30 years or older. A lower mean receptive vocabulary age-equivalent score was reported by Iacono et al. using the PPVT-III (5.2 years) [13]. However, 18% of this sample were reported to have diagnosed or suspected dementia, and so comparison between these and the above studies (which did not include people with dementia) is problematic.

#### Standardised test scores

Standardised IQ test scores were reported by seven identified studies. Full IQ scores (including both verbal and non-verbal subscales) included a median IQ of 41 [17] (Spanish version of KBIT), a mean IQ of 46.6 (Stanford Binet 5th Edition) [15], a mean IQ of 49.7 (Portuguese version of WAIS-III) [18], and mean IQs of 49.7, 48.8, and 33.2 for different age groups of adults with DS (WAIS-R) [8]. Standardised mean verbal IQ scores included 53.4, 51.6, and 33.6 for these age groups using the WAIS-R [8], with other studies reporting standardised mean verbal IQ scores of 52.3 (Portuguese version of WAIS-III; [18]) and 56.6 (PPVT-IV; [26]). Standardised mean non-verbal IQ test scores included 39.3 [12] (brief Leiter-R), 39.0 [10] (full Leiter-R), and 50.8 [18] (Portuguese version of WAIS-III), with Ghezzo et al. reporting 51.4, 52.9, and 36.2 for their different age groups [8].

It is important to note that the lowest full IQ score obtainable on the Leiter-R is 36, and the Stanford Binet 4th Ed supports calculation of IQ scores lower than 40, whereas the lowest full IQ score for the KBIT and WAIS-II are 40 and 45, respectively. It is therefore possible the results reported here are influenced by differing floor levels between tests. Furthermore, floor effects may substantially influence mean test scores. Apart from the high floor effect in standardised IQ tests, it is also worthwhile noting that standardised scoring may result in inflated estimates of true abilities near floor levels, which may differ between tests [27].

#### Raw test scores

Raw tests scores are only useful to compare between studies when the same test has been used. The KBIT-2 has been used in more than one study [11, 16]. These two papers are published by one group and it should be noted that although there is no overlap in data, there is some overlap between participants (31 individuals from Sinai et al. were later recruited by Startin et al.).

**Fig. 2:**
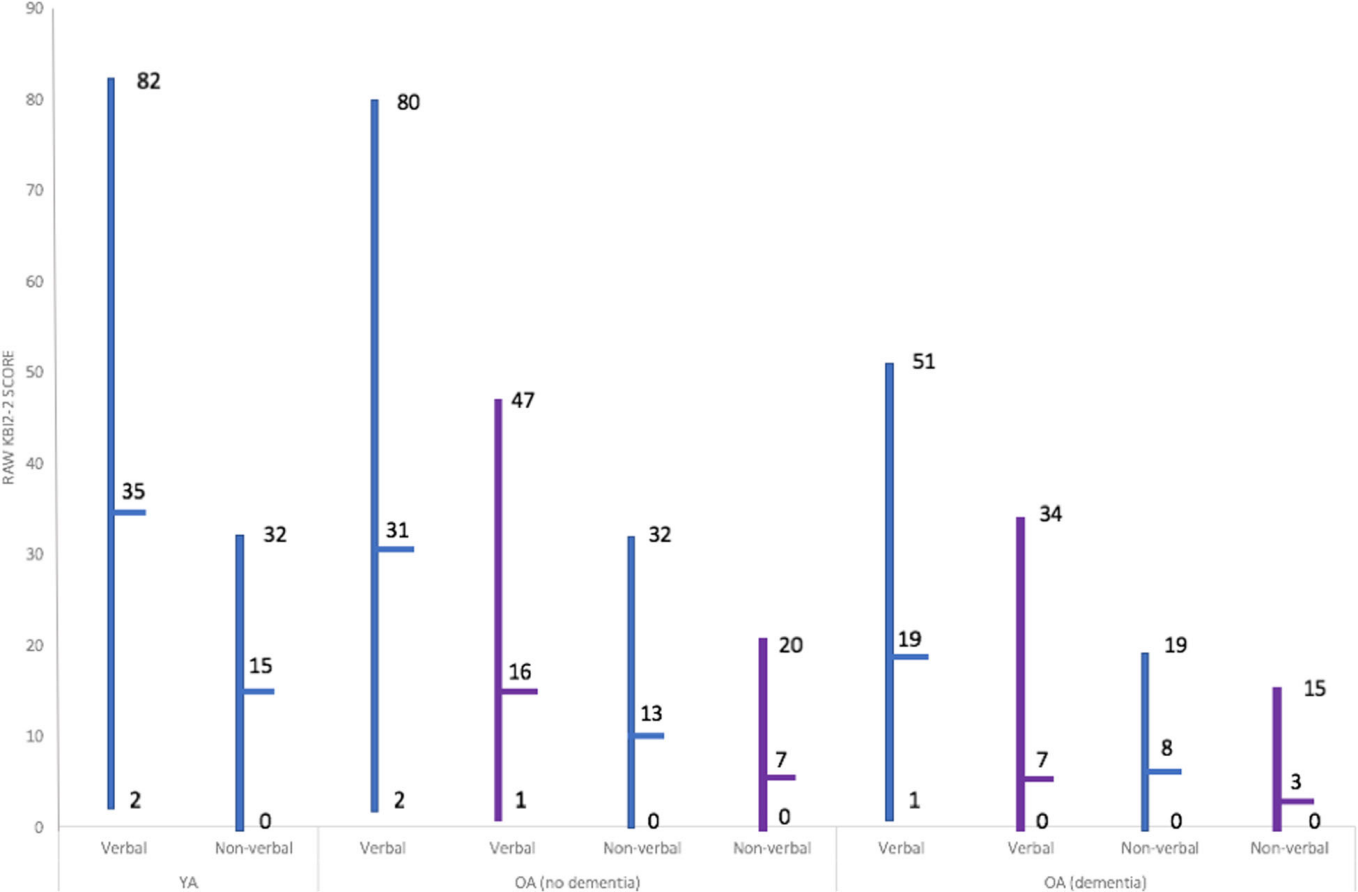
KBIT-2 performance. KBIT-2 raw score ranges and means by participant group (younger adults (YA), older adults without dementia (OA-ND) and older adults with dementia (OA-D)) for Startin et al. (blue) [16] and Sinai et al. (purple) [11]. Subscale differences between each participant group are illustrated

Both studies found a wide range of raw scores for both subscales of the KBIT-2 (see Fig. 2). When examining scores across participant groups (younger adults (YA), older adults without dementia (OA-ND), and older adults with dementia (OA-D)), verbal and non-verbal subscale means and ranges reported by Startin et al. [16] appear relatively similar between YA and OA-ND but were lower in OA-D. Sinai et al. [11] also reported similar reductions in verbal and non-verbal mean scores and ranges between OA-ND and OA-D (YA not included in this study). Overall, these studies demonstrate that raw KBIT-2 scores can be obtained from a range of individuals with DS, including many individuals with dementia.

Raw scores from the WISC-R have been used in two studies [24, 25]. Neither study split participants by age and only included individuals with no decline; therefore, raw test scores between groups cannot be compared. However, Kittler et al. [24] used these scores to explore sex differences in DS and reported females performed significantly better than males on the coding subtest (part of the non-verbal IQ subscale).

## AB scales in people with DS

### Tests

Eleven studies using AB scales in DS were identified for inclusion in this review (see Table 2). A total of 848 participants took part in the studies, ranging from 16 to 71 years old. The only AB scales to be used by more than one study were the Vineland Adaptive Behaviour Scale (VABS) and the second edition of this scale (VABS-II).

**Table 2:** Summary of studies using adaptive ability tests in adults with DS. NR indicates “not reported.” IQ test results not shown (see Table 1)

Study	AB scale	Score type(s) provided	Participants	Participant ages	Raw scores	Standardised scores	Floor effects
Gilmore et al., [28]	Vineland ABS 2nd Edition (VABS-II)	Adaptive Behavior Composite score	21	24.83 (1.20)	NR	51.86 (15.29)	NR
Hartley et al. [14]*	Vineland ABS 2nd Edition (VABS-II)	Adaptive Behavior Composite score	58	37.6 (6.8; ≥ 30)	NR	183.67 (47.65)	NR
Tomaszewski et al., [15]	Vineland ABS 2nd Edition (VABS-II)	Adaptive Behavior Composite score and three individual subscale scores	31	25.9 (5.92)	NR	52.6 (15.8) (Adaptive Behavior Composite); 45.7 (22.2) (Communication Standard Score); 55.5 (14.4) (Daily Living Skills Standard Score); 64.6 (13.7) (Socialisation Standard Score)	NR
Dressler et al. [22]	Vineland ABS (Italian version)	Age equivalent scores	49	28.8 (8.4; 19–52)	NR	Total, 7.26 (3.35; 3.06–10.0); Communication sub-domain, 7.18 (3.51); Daily living skills sub-domain, 7.36 (2.62); Socialisation sub-domain, 7.62 (4.32)	NR
Ghezzo et al. [8]	Vineland ABS		36 adults with DS (of a larger sample of 67 participants which included children)	18–29 years.: *n* = 24, 22.34 (3.40) 30–39 years.: *n* = 17, 34.27 (3.04) ≥ 40: years. *n* = 18, 49.34 (6.91)		The following scales (including subscales) for each of the three adult age groups: Communication, Daily living skills, Socialisation, Motor skills. See paper for details.	
Kishnani et al., [29]**	Vineland ABS	“Composite Supplemental Norm Score”	53 (donepezil group); 59 (placebo group)	24.2 (5.1; 18–36) (donepezil group); 26.0 (5.5; 18–38) (placebo group)	NR	57.4 (10–99) (donepezil group at baseline); 64.1 (30–99) (placebo group at baseline)	NR
Witts and Elders [30]	Vineland ABS	Age equivalent scores	33	36 (8.9; 22–53)	NR	8.5 (3.7; 3.67–18.5)	NR
Kay et al. [19]	ABS	Raw scores	85	38.2	157.5 (59.8; 34–270) Median, 165	NR	NR
de Sola et al. [17]	ABAS-II	Raw scores	86	23.3 (4.3; 16–34)	Total, 635.9 (90.9; 220–627); Communication sub-domain, 80.3 (12.7); Community Use sub-domain, 61.5 (13.2); Functional Academics sub-domain, 67.4 (17.9); Home Living sub-domain, 64.3 (12.1); Health and Safety sub-domain, 57.8 (9.5); Leisure sub-domain, 68.2 (11.5); Self-care sub-domain, 87.5 (8.3); Self-direction sub-domain, 73.5 (14.7); Social Skills sub-domain, 75.3 (14.7)	NR	No subscales showed floor effects
Strydom et al. [23]	ABAS	Raw scores	32	32.59 (6.78; 18–45)	377 (139.53; 98–589)	NR	NR
Startin et al. [16]	Short-ABS	Raw scores	130 aged 36+ years without dementia	47.77 (7.01; 36–71)	Total, 71.89 (23.39; 14–111); SABS P, 26.74 (6.07; 0–33); SABS C, 24.57 (12.06; 0–47); SABS PS, 20.78 (6.97; 3–32)	NR	Total, 0%; SABS P, 0.9%; SABS C, 0.9%; SABS PS, 0%
			51 aged 36+ years with dementia	54.20 (6.95; 38–67)	Total, 42.23 (24.51; 3–92); SABS P, 17.02 (9.70; 0–33); SABS C, 10.00 (15.00; 0–31); SABS PS, 13.00 (11.00; 1–28)	NR	Total, 0%; SABS P, 2.3%; SABS C, 2.3%; SABS PS, 0%
			124 aged 16–35 years	25.24 (5.53; 16–35)	Total, 79.03 (19.73; 28–112); SABS P, 28.91 (4.55; 14–33); SABS C, 27.74 (10.36; 4–47); SABS PS, 22.53 (6.49; 7–32)	NR	Total, 0%; SABS P, 0%; SABS C, 0%; SABS PS, 0%

*Only T1 data used in this review

**Only baseline data used in this review

The Vineland Adaptive Behaviour Scale (VABS [31]) was used by Witts and Elders [30], Kishnani et al. [29], and Ghezzo et al. [8], while Dressler et al. [22] used an Italian version of this scale [32]. The VABS-II was used by Hartley et al., Gilmore and Cuskelly, and Tomaszewski et al. [14, 15, 28]. The Adaptive Behaviour Assessment System (ABAS [33]) was used by Strydom et al. [23], and de Sola et al. [17] used the second edition of this scale (ABAS-II [34]). The Adaptive Behaviour Scale (ABS [35]) was used by Kay et al. [19], and the Short Adaptive Behaviour Scale (SABS [36]), adapted from a later version of the ABS [37], was used by Startin et al. [16].

### Floor effects

Two studies reported floor effect data for the AB scale used. Using raw scores, Startin et al. [16] reported total SABS scores had no floor effects in any group of participants investigated (YA, OA-ND, OA-D). However, when split into its 3 subscales, small floor effects were found. In participants aged 36+ without dementia, floor effects were found in the personal self-sufficiency and community self-sufficiency domains (0.9% for both). For participants aged 36+ with dementia, floor effects were also found in the same two domains (2.3% for both). Kishnani et al. [29] reported no participants were at floor on the VABS. Participants in this study were aged 18–38 and did not have dementia.

### Comparisons between AB scales

Two studies reporting age-equivalent scores from different versions of the VABS found similar mean age-equivalent scores. This was reported as 8.5 years and 7.3 years for Witts and Elders [30] and Dressler et al. [22], respectively. Minimum age-equivalent scores between these two studies were also similar (3.1 years and 3.7 years); however, maximum scores differed (10.0 years and 18.5 years). Kishnani et al. [29] reported mean Composite Supplemental Norm Score from the VABS. The results of these this study are therefore not comparable to those of Witts and Elders [30] and Dressler et al. [22]. The three studies using the VABS-II all reported mean Adaptive Behavior Composite scores of 51.86 [28], 52.6 [15], and 183.67 [14]. It is likely the latter of these scores is greater because for this study participants were required to have a mental age of above 2.5 in addition to at least minimal verbal communication, whereas the former two studies had no such inclusion criteria.

Two identified studies reported raw scores of different versions of the ABAS. de Sola et al. [17] found an overall mean test score of 636 (91 SD) and a range from 220 to 627 using the ABAS-II. In contrast, Strydom et al. [23] reported a mean raw ABAS score of 377 (140 SD) and a range of 98–589 (for further details see Table 2).

### Comparisons between IQ tests and AB scales

de Sola et al. [17] analysed the association between IQ and AB using standardised KBIT IQ scores and raw ABAS-II scores. A significant difference was found between participants with an IQ above and below 40 for most functional skill areas assessed by the ABAS-II, in addition to ABAS-II total score (mean group difference for total ABAS-II score 63.4; *p* = 0.001). This suggests that participants with DS with a higher IQ may have greater competence in daily living and also demonstrates a potential relationship between IQ and AB scales in adults with DS.

AB scales may also correlate with performance on other tests of IQ. Using raw scores of the PCFT and the ABS, Kay et al. [19] noted a highly significant correlation between these two tests (*r* = 0.87; *p* < 0.001). This study provides further evidence that AB and IQ may be related in adults with DS.

In contrast to the findings of these two studies, Dressler et al. [22] found no association between AB and IQ. In this study, IQ tests (either RCPM or the Leiter-R) were used to classify participants by level of ID (mild, moderate, or severe), and VABS scores (Italian version) were compared between groups. No statistically significant differences in VABS scores were observed between groups. However, it is of note that VABS raw scores were not used in this analysis. Instead, VABS raw scores were categorised on an individual basis as above average, average, or below average, relative to mean VABS score for each group. It is possible this approach to analysis prevented the detection of a significant difference in AB scores between groups.

## Discussion

We aimed to provide a systematic review of the literature regarding tests of IQ and AB used in adults with DS, in order to make recommendations regarding the use of such tests with this population. Overall a wide variety of different IQ tests and AB scales were identified, with a wide range of differing score types provided (including raw scores, age-equivalent scores, full IQ scores, verbal IQ scores, and non-verbal/performance IQ scores). Studies largely differed in criteria for participant inclusion (e.g. only those able to complete tests) and in the reporting of test results by sub-populations. There was also little overlap in the tests used between studies. Together, these factors make the comparison of tests between studies problematic.

Where reported, floor effects for IQ tests were particularly high for standardised test scores. Floor effects for raw total BPVS-II scores and raw WISC-R sub-scores were moderate (around 9%) and high (around 50%), respectively. In contrast, floor effects reported for KBIT-2 raw scores were minimal. Verbal raw KBIT-2 scores were particularly low (including for participants with dementia). The number of participants at floor for AB scales was only reported by one study [16]. This study found no floor effects using total raw SABS scores (including for individuals with dementia); however, when subdomains of this test were examined, small floor effects were seen for two out of three subdomains. Further, although Kay et al. [19] did not explicitly report floor effects using the ABS, the authors noted that floor effects were less marked on this scale compared to the IQ test used in the study (the PCFT). Together, these findings indicate that raw KBIT-2 scores and raw AB scores may be particularly suited to tracking longitudinal change in adults with DS, due to minimal floor effects on these measures prior to the onset of cognitive decline.

Although it appears that raw scores may benefit from reduced floor effects compared to standardised scores, it should be noted that the use of raw scores has various limitations. This includes the inability to directly compare level of functioning to that of the TD population (in contrast to the use of standardised or age-equivalent IQ scores, through which this is inherently possible). Additionally, the clinical significance of differences in raw score values both between and within individuals over time has not yet been established.

In some studies, child versions of IQ tests (e.g. WISC instead of WAIS) have been used in adults with DS [21, 24, 25]. While this might limit floor effects and should therefore be more sensitive to differences in performance, age-adjusted IQ norms are only available for children, and therefore only age-equivalent or raw scores can be used in adults. Age appropriateness could also be an issue. The generalisability of IQ tests and AB scales in general is an important issue that warrants further investigation. Specifically, the tests identified here were developed in Western populations, and most were developed for use in TD individuals.

Many IQ tests identified in this review are dependent on language. Significant relative weaknesses in language are a characteristic feature of the cognitive profile for individuals with DS [38, 39] (see review by Silverman [6]). The use of language-based IQ tests in this population is therefore problematic as specific deficits in language may mask the true level of individuals’ general ability and skew group test results. Accordingly, some studies identified in this review excluded participants without sufficient verbal skills. Non-verbal/performance subscales on IQ tests are less likely to be substantially influenced by language and so may be more appropriate for use in this population. However, studies utilising these subscales have reported higher floor effects compared to verbal subscales [11, 16]. It is also worthwhile noting that IQ tests with language as an integral component require substantial translation and subsequent revalidation for use in different language-speaking populations. For larger international studies, translation into different languages is a particular barrier, and so non-verbal/performance tests may be preferable to verbal tests. Future research could explore the use of simple non-verbal/performance tests that could be used in people with DS with lower floor effects, though it will need to be established if this would over-estimate IQ.

In this review, “floor” refers to the lowest possible score obtainable on a particular test. However, it may be more appropriate to discuss floor effects in reference to the lowest score below which a decline of significance cannot be detected, for example, two standard errors of the mean (SEM) above the lowest score. Floor effects discussed in this review may therefore be underestimated.

It is likely other IQ tests exist that may be suitable for adults with DS but were not utilised by any studies identified in this review. In particular, d’Ardhuy et al. [10] suggested the Leiter-III may be a more appropriate standardised IQ test for use in people with DS compared to the Leiter-R. This is based on a clinical trial of 180 individuals with DS (Clinical.Trials.gov identifier NCT01920633) which reported a floor effect of only 1% with this IQ test [10]. Furthermore, the potential utility of the Stanford-Binet IQ test in individuals with DS has been highlighted by other studies that did not meet inclusion criteria of this review. For example, Silverman et al. [40] demonstrated a strong linear correlation between IQ score measured on the Stanford-Binet and the WAIS (*r* = .818) in individuals with an ID (70.3% with DS), confirming that the two scales measured the same underlying construct(s). However, IQ estimates using the WAIS were consistently higher in this study, and more than 85% of individuals with DS had IQ scores that were more than 10 points higher on the WAIS compared to the Stanford-Binet, indicating direct comparison of standardised IQ scores between these two tests requires further validation. Future research should further explore the use of these IQ tests in adults with DS.

With regards to AB measures, three measures were commonly used: the Vineland adaptive behavior scales, the Adaptive Behavior Scale (ABS, including its short form), and the Adaptive Behaviour Assessment System (2nd edition; ABAS-II). These measures did not have significant floor effects and in two identified studies showed a correlation with IQ test scores [17, 19]. This suggests that AB measures are a useful addition to research studies of cognitive abilities in individuals with DS alongside IQ testing and may allow for an assessment of general ability in individuals with DS who cannot engage with IQ tests or who are at floor for IQ tests.

AB measures may represent a broader construct compared to IQ and are likely to be influenced by an individual’s physical abilities as well as their training and support to maintain independence. Nevertheless, the studies reviewed here demonstrated that AB measures can be useful in tracking change in general abilities over time, and showed significant differences in scores between groups defined by age or dementia status. Further research is required to demonstrate the relationship between different subscales of AB measures such as the VABS and IQ scores, and between different AB scales. The particular strengths and weaknesses of AB domains in DS should be established, and the development of shorter versions of AB measures will be desirable.

## Conclusions

Recommendations following this review have been summarised in Table 3. The main recommendations are that the use of raw scores for certain IQ tests such as the K-BIT2 can minimise floor effects and may therefore be particularly useful in longitudinal studies, though it must be acknowledged that the significance of changes in raw scores are currently uncertain. The use of more common IQ tests (e.g. KBIT, BPVS, WISC-R, RCPM) and AB tests (e.g. VABS, ABS, ABAS) should be encouraged more broadly in both research and clinical settings while the use of non-verbal/performance IQ tests may be preferable in multi-site international studies involving populations speaking different languages. Finally, studies may benefit from the use of both IQ and AB scales, particularly if participants include individuals with a broad range of abilities.

**Table 3:** Recommendations for future studies of adults with DS and for the DS research community

Recommendations for individual studies of adults with DS	
1. The use of raw scores for certain IQ tests, particularly the K-BIT2, can minimise floor effects and may therefore be particularly useful in longitudinal studies to track change in cognitive ability over time.	
2. Non-verbal/performance IQ tests may be useful in multi-site international studies involving populations speaking different languages.	
3. The use of more common IQ tests (e.g. KBIT, BPVS, WISC-R, RCPM) and AB tests (e.g. VABS, VABS-II, ABS, ABAS) should be encouraged more broadly in both research and clinical settings. Practical implications of this are extremely valuable for detecting changes in ability.	
4. Studies may benefit from the use of both IQ and AB scales, particularly if participants include individuals with a broad range of abilities.	
Recommendations for the DS research community	
1. The development of reporting standards would increase the ability of different study findings to be compared, for example reporting both raw and standardised scores, full floor effects, and separately reported results for individual DS subpopulations.	
2. Sharing of data from published studies would allow comprehensive comparison between different IQ tests and between different AB tests, in addition to correlations between these two measures for different DS subpopulations.	

It is also apparent from this review that there is likely a wealth of raw IQ and AB test data that has not been included in the studies identified here. Furthermore, it is apparent that a potential limitation of the current research field is that many studies do not exclude (or analyse separately) individuals with cognitive decline or dementia, or individuals with a non-trisomy 21 form of DS. The research community may therefore benefit from an effort to share such data in order to make full and valid comparisons between scales and between different subpopulations of individuals with DS. Such information is likely to be of benefit to both clinicians and researchers.

## Data Availability

Data sharing is not applicable to this article as no datasets were generated or analysed during the current study.

## Appendix

**Table 4:** Summary of intelligence and adaptive ability tests used by identified studies in adults with DS

	Test name	Authors	Domains measured
Intelligence tests	The British Picture Vocabulary Scale (2nd edition); BPVS-II	Dunn et al. [41]	Verbal IQ (receptive language)
	The Kaufman Brief Intelligence Test; KBIT	Kaufman and Kaufman [42]	Verbal and non-verbal IQ
	The Kaufman Brief Intelligence Test (2nd edition); KBIT-2	Kaufman and Kaufman [43]	Verbal and non-verbal IQ
	The Leiter International Performance Scale-Revised; Leiter-R	Roid and Miller [44]	Non-verbal IQ
	The Matrix Analogies Test-Expanded Form; MAT	Naglieri [45]	Non-verbal IQ
	The Peabody Picture Vocabulary Test (3rd edition); PPVT-III	Dunn and Dunn [46]	Verbal IQ (receptive language)
	The Peabody Picture Vocabulary Test (4th edition); PPVT-IV	Dunn and Dunn [47]	Verbal IQ (receptive language)
	The Revised Peabody Picture Vocabulary Test; PPVT-R	Dunn and Dunn [48]	Verbal IQ (receptive language)
	The Prudhoe Cognitive Function Test; PCFT	Kay et al. [19]	General cognitive functioning
	Raven’s Coloured Progressive Matrices; RCPM	Raven [49]	Non-verbal IQ (abstract reasoning)
	Stanford Binet 5th edition; SB-5	Roid [50]	Verbal and performance IQ
	The Wechsler Adult Intelligence Scale (3rd edition); WAIS-III	Wechsler [51]	Verbal and performance IQ
	The Wechsler Adult Intelligence Scale Revised; WAIS-R	Wechsler [52]	Verbal and performance IQ
	The Wechsler Intelligence Scale for Children-Revised; WISC-R	Wechsler [53]	Verbal and performance IQ
	The Wechsler Preschool and Primary Scale of Intelligence – Revised; WPPSI-R	Wechsler [54]	Verbal and performance IQ
	The Woodcock–Johnson Tests of Cognitive Abilities; WJTCA-R	Woodcock [55]	General cognitive functioning
Adaptive ability tests	Adaptive Behaviour Assessment System; ABAS	Harrison and Oakland [33]	Adaptive abilities (main domains include: Conceptual, Social and Practical)
	Adaptive Behaviour Assessment System (2nd edition); ABAS-II	Harrison and Oakland [34]	Adaptive abilities (main domains include: Conceptual, Social and Practical)
	Short Adaptive Behaviour Scale; SABS	Hatton et al. [36]	Adaptive abilities (main domains include: Personal Self-Sufficiency, Community Self-Sufficiency, Personal-Social Responsibility)
	Vineland Adaptive Behaviour Scale; VABS	Sparrow et al. [31]; Italian version Balboni and Pedrabissi [32]	Adaptive abilities (main domains include: Communication, Daily Living Skills, Socialisation, Motor Skills)
	Vineland Adaptive Behaviour Scale 2nd Edition; VABS-II	Sparrow et al. [56]	Adaptive abilities (main domains include: Communication, Daily Living Skills, Socialisation, Motor Skills)

## Abbreviations

AB: Adaptive behaviour
ABAS: Adaptive Behaviour Assessment System
ABAS-II: Adaptive Behaviour Assessment System 2nd edition
ABS: Adaptive Behaviour Scale
BPVS-II: The British Picture Vocabulary Scale 2nd edition
IQ: Intelligence quotient
KBIT: Kaufman Brief Intelligence Test
KBIT-2: Kaufman Brief Intelligence Test 2nd edition
Leiter-r: Leiter International Performance Scale Revised
MAT: Matrix Analogies Test-Expanded Form
OA-D: Older adults with dementia
OA-ND: Older adults without dementia
PCFT: Prudhoe Cognitive Function Test
PPVT-III: Peabody Picture Vocabulary Test 3rd edition
PPVT-IV: Peabody Picture Vocabulary Test 4th edition
PPVT-R: Revised Peabody Picture Vocabulary Test
RCPM: Raven’s Coloured Progressive Matrices
SABS: Short Adaptive Behaviour Scale
SEM: Standard errors of the mean
VABS: Vineland Adaptive Behaviour Scale
VABS-II: Vineland Adaptive Behaviour Scale 2nd Edition
WAIS-III: Wechsler Adult Intelligence Scale 3rd edition
WAIS-R: Wechsler Adult Intelligence Scale – Revised
WISC-R: Wechsler Intelligence Scale for Children – Revised
WJTCA-R: Woodcock–Johnson Tests of Cognitive Abilities
WPPSI-R: Wechsler Preschool and Primary Scale of Intelligence – Revised
YA: Younger adults
